# Preoperative assessment of fistula-in-ano using SonoVue enhancement during three-dimensional transperineal ultrasound

**DOI:** 10.1093/gastro/goae002

**Published:** 2024-02-27

**Authors:** Jun Yang, Qing Li, Hua Li, Heng Zhang, Donglin Ren, Zhiyi Zhang, Dan Su, Haihua Qian

**Affiliations:** Department of Anorectal Surgery, The Affiliated Hospital of Nanjing University of Chinese Medicine, Nanjing, Jiangsu, P. R. China; Department of Anorectal Surgery, Qilu Hospital (Qingdao), Cheeloo College of Medicine, Shandong University, Qingdao, Shandong, P. R. China; Department of Anorectal Surgery, The Affiliated Hospital of Nanjing University of Chinese Medicine, Nanjing, Jiangsu, P. R. China; Department of Anorectal Surgery, Xingtai People’s Hospital, Hebei Medical University, Xingtai, Hebei, P. R. China; Department of Surgical Oncology, Xingtai People’s Hospital, Hebei Medical University, Xingtai, Hebei, P. R. China; Department of Colorectal and Anal Surgery, The Sixth Affiliated Hospital, Sun Yat-sen University, Guangzhou, Guangdong, P. R. China; Guangdong Provincial Key Laboratory of Colorectal and Pelvic Floor Diseases, The Sixth Affiliated Hospital, Sun Yat-sen University, Guangzhou, Guangdong, P. R. China; Biomedical Innovation Center, The Sixth Affiliated Hospital, Sun Yat-sen University, Guangzhou, Guangdong, P. R. China; Department of Colorectal and Anal Surgery, The Sixth Affiliated Hospital, Sun Yat-sen University, Guangzhou, Guangdong, P. R. China; Guangdong Provincial Key Laboratory of Colorectal and Pelvic Floor Diseases, The Sixth Affiliated Hospital, Sun Yat-sen University, Guangzhou, Guangdong, P. R. China; Biomedical Innovation Center, The Sixth Affiliated Hospital, Sun Yat-sen University, Guangzhou, Guangdong, P. R. China; Department of Ultrasound in Medicine, Qilu Hospital (Qingdao), Cheeloo College of Medicine, Shandong University, Qingdao, Shandong, P. R. China; Department of Colorectal and Anal Surgery, The Sixth Affiliated Hospital, Sun Yat-sen University, Guangzhou, Guangdong, P. R. China; Guangdong Provincial Key Laboratory of Colorectal and Pelvic Floor Diseases, The Sixth Affiliated Hospital, Sun Yat-sen University, Guangzhou, Guangdong, P. R. China; Biomedical Innovation Center, The Sixth Affiliated Hospital, Sun Yat-sen University, Guangzhou, Guangdong, P. R. China; Department of Anorectal Surgery, The Affiliated Hospital of Nanjing University of Chinese Medicine, Nanjing, Jiangsu, P. R. China

**Keywords:** fistula-in-ano, transperineal ultrasound, SonoVue, enhancement

## Abstract

**Background:**

Accurate preoperative evaluation of fistula-in-ano can guide the choice of surgical procedure and may improve healing rates. This prospective study aimed to evaluate the accuracy of conventional 3D transperineal ultrasound (3D-TPUS) compared with SonoVue (SVE)-enhanced 3D-TPUS for the detection and classification of anal fistula.

**Methods:**

In this prospective study, 3D-TPUS reconstructions were performed before and after SVE enhancement in 60 patients with fistula-in-ano who intended to undergo surgery at the Department of Anorectal Surgery, Qilu Hospital, Cheeloo College of Medicine, Shandong University (P. R. China) between January 2021 and October 2021. Accuracies of anal fistula classification, complexity classification, detection of anal fistula branches, and detection of internal opening between 3D-TPUS and SVE 3D-TPUS were compared based on a reference standard—intraoperative findings.

**Results:**

This study enrolled 60 patients (mean age, 37.1 ± 11.4 years; mean follow-up, 9 ± 3 months). Intraoperative findings showed that the fistula type was intersphincteric in 23 patients (38.3%), trans-sphincteric in 35 (58.3%; 12 high and 23 low), and suprasphincteric in 2 (3.3%). Moreover, 68 internal openings were found. Compared with the accuracy of 3D-TPUS, that of SVE 3D-TPUS was similar in fistula classification [95.0% (57/60) vs 96.7% (58/60), *P *=* *0.392], but significantly higher in internal opening evaluation [80.9% (55/68) vs 97.1% (66/68), *P *=* *0.001], complexity classification [85.0% (51/60) vs 98.3% (59/60), *P *=* *0.018], and detection of fistula branches [70.4% (19/27) vs 92.6% (25/27), *P *=* *0.031].

**Conclusions:**

SVE 3D-TPUS may be a useful examination for patients with perianal fistulae because of its high accuracy and consistency with intraoperative findings, especially in complex fistula-in-ano and difficult cases.

## Introduction

Fistula-in-ano is a tract that connects the perineal skin to the anal canal [1]. Its annual incidence is approximately two cases per 10,000 population, and it affects men more than women [2]. Surgery remains the mainstay of treatment of non-Crohn’s anal fistula, with the aim of curing the fistula while preserving anal sphincter function [3]. However, anal fistula recurrence after surgery seems to be an unresolved issue. The reported rate of recurrence after anal fistula surgery was 3%–57%, with varying rates among different procedures [4]. Therefore, accurate preoperative evaluation of anal fistula is crucial to obtain the best surgical outcome. It can help doctors select the correct surgical procedure with the lowest recurrence rate. Transperineal ultrasound (TPUS) is an effective, inexpensive, safe, and readily available method associated with minimal pain, mainly because it is non-invasive and does not need a special application set or probe. However, it is a rarely used diagnostic tool in daily practice [5, 6]. TPUS assessment of perianal lesions can be appropriately improved by using color Doppler, contrast-enhancement, 3D imaging, and sonoelastography [7]. To date, only a few pieces of research have evaluated the accuracy of 3D-TPUS in patients with anal fistulae. Therefore, we evaluated the accuracy of SonoVue (SVE) enhancement during 3D-TPUS in detecting and classifying anal fistulae, using intraoperative findings as the reference standard.

## Patients and methods

### Patients

This prospective study enrolled patients who intended to undergo surgery at the Department of Anorectal Surgery, Qilu Hospital (Qingdao), Cheeloo College of Medicine, Shandong University (Shandong, P. R. China) between January 2021 and October 2021. The studies involving human participants were reviewed and approved by the ethics committee of the Qilu Hospital of Shandong University (approval code: KYLL-KS-2021055). The patients provided written informed consent to participate in this study.

#### Inclusion criteria

Inclusion criteria were as follows: (i) age of >18 years; (ii) diagnosed with anal fistula; (iii) underwent 3D-TPUS and SVE 3D-TPUS after admission; (iv) gave informed consent; and (v) underwent surgery, with an elaborate operative report, within 24 h after 3D-TPUS.

#### Exclusion criteria

Exclusion criteria were as follows: (i) acute diarrhea or perianal eczema; (ii) severe cardiovascular or cerebrovascular disease; (iii) severe liver or kidney dysfunction; (iv) inflammatory bowel disease or intestinal tuberculosis or malignant anal tumors; (v) allergy to ultrasound contrast agents; (vi) lack of obvious external opening; and (vii) presence of acute infection in perianal area.

### Instruments and examination methods

**Figure 1.**
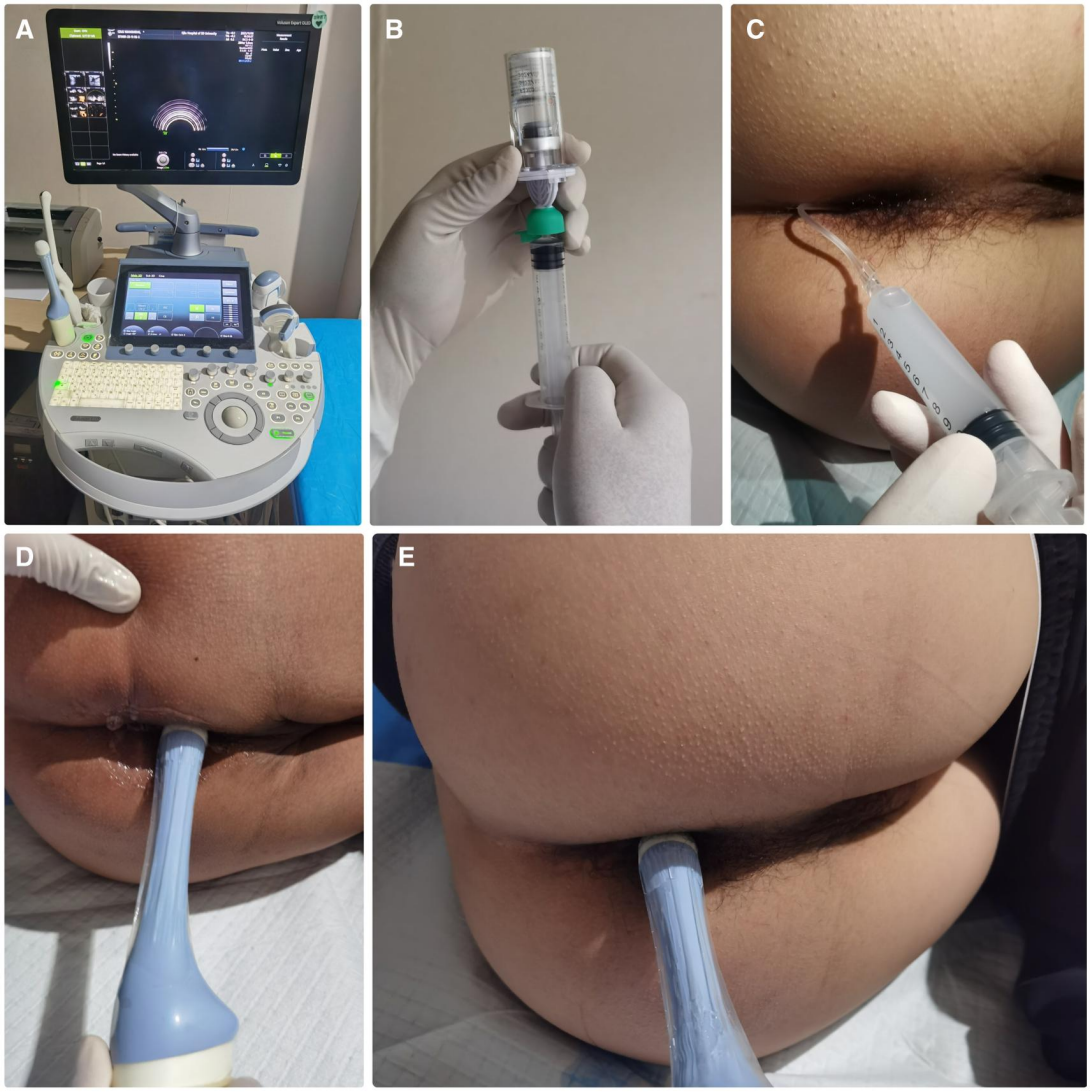
SonoVue enhancement during 3D transperineal ultrasound. (A) GE Voluson E10 diagnostic ultrasound system. (B) Proportioning SonoVue suspension. (C) Injecting SonoVue suspension. (D) Transperineal ultrasound inspection (the probe was placed above the perineal body). (E) Transperineal ultrasound inspection (the probe was placed above the anus).

All patients underwent preoperative 3D-TPUS in the left lateral position. A GE Voluson E10 diagnostic ultrasound system with a 180° rotating 3D volume probe (frequency 5–9 MHz) was used. Sagittal, coronal, and transverse images were acquired and recorded as an anorectal image cube in a video format. Two sets of image cubes were acquired before and after injection of SVE. We placed a plastic venous cannula ∼5–10 mm into the external fistulous opening. Subsequently, an appropriate volume of SVE suspension (Bracco Company, Italy) was injected through the cannula to observe for the presence of a hyperechoic fistula tract and to locate the internal opening. The area was gently kneaded to facilitate the movement of the SVE suspension within the fistula tract (Figure 1). Two experienced doctors reviewed the unenhanced and enhanced image cubes, respectively, without knowledge of the patient’s clinical data.

### Evaluation criteria

**Figure 2.**
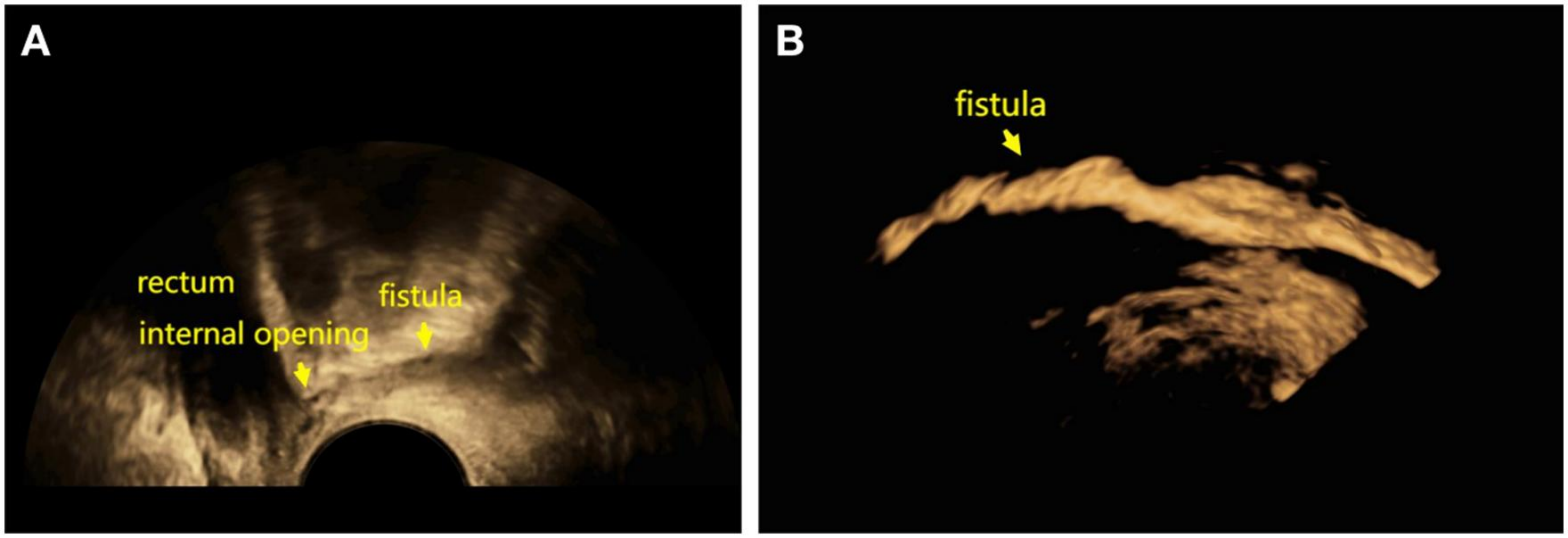
Fistula imaging under ultrasound. (A) The fistula is seen as a linear hypoechoic tract. (B) The fistulous tract turns lustrously bright and reflective after injection of SonoVue suspension.

**Figure 3.**
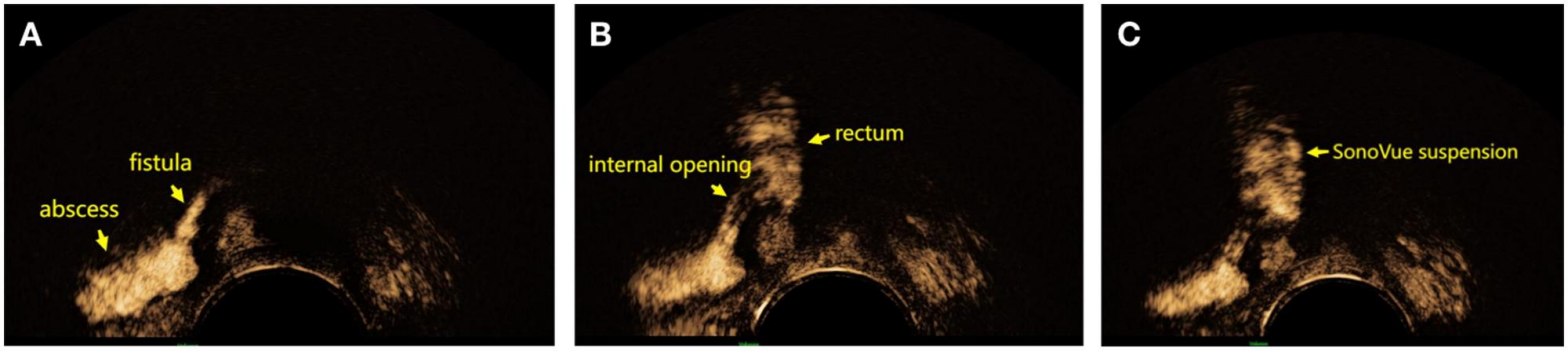
After rupture of the mucous membrane, SonoVue suspension is seen to flow into the rectal cavity

Diagnosis of anal fistula using SVE 3D-TPUS was based on the presence of (i) linear or branched hypoechoic fistulous tracts, which might contain small gas bubbles and turn brightly reflective after injection of SVE suspension (Figure 2), and (ii) an internal opening, seen as a disruption in the hypoechoic ring of the internal anal sphincter and sub-epithelial tissue. The SVE suspension flowed into the rectal cavity after rupture of the mucous membrane (Figure 3).

### Classification criteria

#### Park’s classification

The anal fistula was classified as follows, based on a previous report [8]: (i) intersphincteric—if the fistula ramified only in the intersphincteric plane; (ii) trans-sphincteric—if the tract passed from the intersphincteric plane through the external sphincter complex at varying levels into the ischiorectal fossa; (iii) suprasphincteric—if the tract passed through the intersphincteric plane over the top of the puborectalis, then downwards again through the levator plate to the ischiorectal fossa, and finally to the skin; and (iv) extrasphincteric—if the tract passed from the perineal skin through the ischiorectal fat and levator muscles, and into the rectum (i.e. entirely outside the external sphincter complex).

#### Complexity assessment

The anal fistula was classified as simple or complex [9]. Simple anal fistulae included intersphincteric and low trans-sphincteric fistulae that involved <30% of the external sphincter. Complex anal fistulae included trans-sphincteric fistulae that involved >30% of the external sphincter and suprasphincteric, extrasphincteric, horseshoe, branching, or anterior fistulae in women.

### Surgery

All patients underwent surgery within 24 h after the 3D-TPUS examination. The intraoperative findings were recorded in detail. Fistulectomy was performed in 46 cases with intersphincteric or low trans-sphincteric fistulae. The ligation of the intersphincteric fistula tract procedure was performed in 2 cases with high trans-sphincteric fistulae and thread-drawing therapy was performed in another 10 cases with high trans-sphincteric fistulae and 2 cases with suprasphincteric fistulae.

### Follow-up

Patients were followed up both 6 and 12 months after surgery. The follow-up included symptoms (purulent discharge, intermittent perianal swelling, pain) and perianal examinations (discharge, external opening, nodule fistula). We defined success as the absence of any discharge and closure of the external opening 6 months after surgery, failure as the persistence of discharge from the external opening following surgery, and recurrence as the reappearance of discharge and the fistula after initial complete wound healing [2]. Rates of healing and recurrence were recorded.

### Statistical analyses

Data were analysed using SPSS version 25.0 (IBM, USA). Categorical data are expressed as numbers (percentages) and were compared using the paired χ^2^ test (McNemar’s test). Using the operative outcomes as the gold standard, the number of fistulae, correct classification of each fistula, and detection of internal opening were compared between 3D-TPUS and SVE 3D-TPUS. *P*-values of <0.05 were considered statistically significant.

## Results

### Patient demographics

A total of 62 patients with fistula were recruited at the beginning of this study. Two patients were excluded—one with tuberculosis and another with ulcerative colitis. The remaining 60 patients were enrolled in this study, which included 42 men and 18 women with a mean age of 37.1 ± 11.4 years (range, 20–72 years). 3D-TPUS and SVE 3D-TPUS were endured well by all patients. All patients completed follow-up. The average follow-up was 9 ± 3 months.

### Anal fistula classification

Based on the intraoperative findings, the type of anal fistula was intersphincteric in 23 patients (38.3%), trans-sphincteric in 35 (58.3%, 12 high and 23 low), and suprasphincteric in 2 (3.3%). The accuracy in classifying the anal fistulae was not significantly different between 3D-TPUS and SVE 3D-TPUS [95.0% (57/60) vs 96.7% (58/60), *χ*^2^ = 3.000, *P *=* *0.392, Table 1].

**Table 1.** Accuracy of 3D-TPUS and SVE 3D-TPUS in 60 patients with anal fistulae

Item	3D-TPUS	SVE 3D-TPUS	Surgical finding
Park’s classification	57 (95.0)	58 (96.7)	60
Intersphincteric	22 (95.7)	23 (100)	23
Trans-sphincteric	34 (97.1)	33 (94.3)	35
Suprasphincteric	1 (50.0)	2 (100)	2
Extrasphincteric	0	0	0
Branches	19 (70.4)	25 (92.6)	27
Complexity classification	51 (85.0)	59 (98.3)	60
Complex	30 (78.9)	37 (97.4)	38
Simple	21 (95.5)	22 (100)	22
Internal opening	55 (80.9)	66 (97.1)	68

3D-TPUS = 3D transperineal ultrasound; SVE 3D-TPUS = SonoVue-enhanced 3D transperineal ultrasound.

### Complexity classification

The accuracy in complexity classification was significantly higher with SVE 3D-TPUS than with 3D-TPUS [85.0% (51/60) vs 98.3% (59/60), *χ*^2^ = 8.000, *P *=* *0.018, Table 1].

### Detection of anal fistula branches

**Figure 4.**
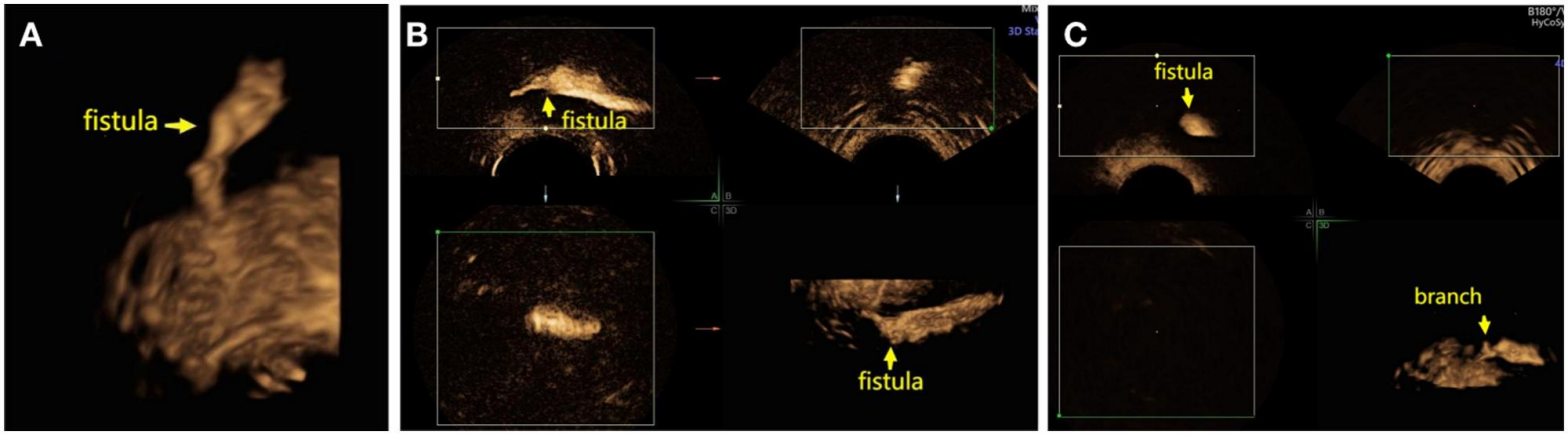
The tomographic ultrasound imaging technique is found to be helpful in tracing the pathway of fistulae. (A) Fistula imaging under SonoVue 3D transperineal ultrasound. (B) Sagittal, coronal, and transverse images of the fistula. (C) Imaging of branch fistula.

During surgery, 27 branches were detected (Figure 4). The number of preoperatively detected fistula branches was significantly higher by SVE 3D-TPUS than by 3D-TPUS [25 (92.6%) vs 19 (70.4%), *χ*^2^ = 6.000, *P *=* *0.031, Table 1].

### Detection of internal opening

**Figure 5.**
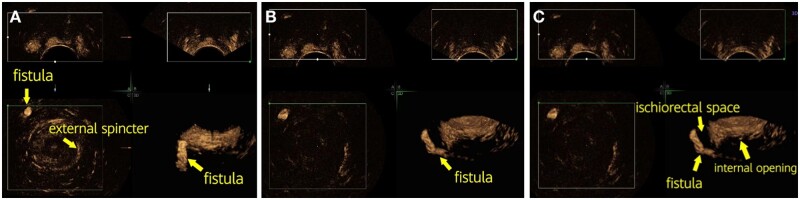
Suprasphincteric fistula and internal opening. (A) Position relationship between anal fistula and sphincter. (B) Fistula imaging under SVE 3D-TPUS. (C) Internal opening and fistula imaging under SVE 3D-TPUS. SVE 3D-TPUS, SonoVue 3D transperineal ultrasound.

Among the 60 patients who underwent surgery, 68 internal openings were found during surgery (Figure 5). The number of preoperatively detected internal openings was significantly higher by SVE 3D-TPUS than by 3D-TPUS [97.1% (66/68) vs 80.9% (55/68), *P *=* *0.001, Table 1]. Compared with 3D-TPUS, SVE 3D-TPUS had a higher accuracy in detecting internal openings in the fistulae.

## Discussion

All patients were followed for 9 ± 3 months. The healing rate was 100% and the recurrence rate was 3.33%. In our study population, the rate of post-operative anal fistula recurrence was consistent with the previously reported recurrence rate of 3%–57% [4], which varied among different procedures. One of them was a 50-year-old female patient with diabetes mellitus. Both 3D-TPUS and SVE 3D-TPUS reported the right anterior high trans-sphincteric anal fistula in the internal sinus at the 1 point at the resection site. A ligation of the intersphincteric fistula tract procedure was performed. The condition recurred 10 months after surgery. Another case was a 36-year-old male patient. 3D-TPUS reported a right high trans-sphincteric anal fistula with abscess with unknown internal opening. Preoperative SVE 3D-TPUS reported the right high trans-sphincteric anal fistula and the internal opening in the 6 point anal gland (lithotomy position). Thread-drawing therapy was performed. The condition recurred 12 months after surgery. In our study, we found that there were many factors that affected the outcomes of anal fistula surgery, including age, diabetes mellitus, surgical procedure, number of fistulas, type of fistula, location of the internal opening, and so on. The primary aim of this study was to explore the value of SVE enhancement during 3D-TPUS in the preoperative assessment of anal fistulae. Our results showed that, compared with 3D-TPUS, SVE 3D-TPUS had higher accuracy in terms of internal opening evaluation, complexity classification, and fistula branch detection. The diagnosis of anal fistulae entails visual inspection, palpation, digital examination, anoscopic examination, barium enema, fistulography, and imaging methods, such as ultrasound, CT, and magnetic resonance imaging (MRI) [10].

The value of preoperative fistula imaging is the detection of tracts and internal openings, which might be missed during surgical exploration [11]. Accurate preoperative imaging and classification of the type of perianal fistula can guide the choice of the appropriate surgical procedure, thereby possibly improving healing rates [12]. MRI and 3D endoanal ultrasound (EAUS) for the diagnosis of anal fistula have been proven to be accurate and reproducible. 3D-EU and MRI are usually the first-line option [2] and are currently the most commonly used imaging techniques for the diagnosis of anal fistulae. The accuracy of MRI in diagnosing perianal fistula is ≥85%. EAUS may be useful in the management of patients with anal abscesses or fistulae and was demonstrated to be concordant with the operative findings in 73%–100% of cases [9]. However, MRI is not applicable in patients with metallic clips or in those with claustrophobia, and EAUS can be painful or not feasible in patients with anal stenosis and cannot assess the pathological changes that extend to the gluteal region [13]. In a 2014 prospective study on 50 patients with suspected anorectal fistulae, Singh *et al.* [14] reported that MRI had a sensitivity of 95%, a specificity of 80%, and a positive predictive value of 97% in detecting and grading the primary fistula tract. However, both MRI and EAUS require specialized and relatively expensive equipment. Furthermore, both are contraindicated or difficult to perform in nervous patients.

Ultrasound has an acceptable diagnostic performance for fistula-in-ano [15]. Compared with EAUS and MRI, TPUS seems to be a simpler, less expensive, easily available, and repeatable method that can adequately evaluate perianal fistulae and abscesses [16] and is often more acceptable for patients. TPUS assessment of perianal lesions can be appropriately improved using 3D imaging [7]. TPUS has been introduced for anal sphincter anatomy on multiple planes and high-resolution imaging of the anal canal [17]. In our study, 3D-TPUS had 95% accuracy in detecting the type of perianal fistula, which was confirmed by the surgical findings. As an ultrasonic contrast medium, hydrogen peroxide had been commonly injected into the external opening of a fistula tract, which enhances because of the formation of hyper-reflective gas bubbles. This was reported to improve the detailed assessment and increase the accuracy of imaging of fistula tracts and internal openings [18–19]. However, the use of hydrogen peroxide to detect a suspected lesion has been debated because it was observed to generate a series of artifacts that may preclude detailed assessment of the area [20]. Moreover, hydrogen peroxide is highly irritating to the mucous membrane and may cause intolerable pain in the anorectal region. However, SVE is safe, rarely causes adverse reactions, does not damage the rectal mucosa, and reduces mucosal stimulation, thereby making the injection site less painful and lowering the sphincter tension [21]. Furthermore, SVE is a second-generation contrast agent, which consists of sulfur hexafluoride-filled microbubbles. It has a strong nonlinear harmonic response when insonated with low acoustic power [22]. When SVE suspension flows into the internal opening along the tract, it creates pressure and opens a partially closed internal opening. In our study, SVE 3D-TPUS was tolerated well by all 60 patients and had a significantly higher accuracy in detecting the location of the internal opening compared with that of 3D-TPUS. Moreover, the tomographic ultrasound imaging technique was found to be helpful in tracing the pathway of the fistulae and internal openings (Figures 4 and 5) and improved the diagnostic accuracy. In this study, the accuracy of complexity classification was significantly higher with SVE 3D-TPUS than with 3D-TPUS. Therefore, routine use of SVE during 3D-TPUS may be recommended for the detection of complex anal fistulae.

Maconi *et al.* [23] reported that TPUS was accurate and comparable with MRI in terms of perianal fistula detection and can, therefore, be used as a first-line modality for the investigation of perianal pathology. A prospective study on 23 patients with Crohn’s disease reported nearly identical diagnostic accuracies between TPUS and MRI using operative findings as the basis [24]. In our study, the accuracy of classifying anal fistulae based on Park’s classification was higher with SVE 3D-TPUS than with 3D-TPUS. A scar and a chronic inflammatory fistula have no obvious difference and are difficult to distinguish on 3D-TPUS. Meanwhile, SVE 3D-TPUS can clearly demonstrate continuous hyperechoic microbubbles in the fistula branches. This can explain our results on the improved accuracy in detecting the number of fistula branches using SVE 3D-TPUS.

Our study had some limitations. The small sample size and the absence of cases with extrasphincteric fistulae could have influenced the results. This can be further addressed by enrolling a larger sample size in future studies.

## Conclusions

Both 3D-TPUS and SVE 3D-TPUS had high accuracy in detecting and classifying fistula-in-ano, but the latter displayed better diagnostic value in detecting internal openings and could provide a distinct preoperative roadmap of fistula tracts, particularly the high trans-sphincteric and suprasphincteric cases. This may be helpful and necessary to prevent recurrence after surgery.

## Authors’ Contributions

J.Y. conceived the study conception, analysed the data, drafted the manuscript, and reviewed the literature; Q.L. conceived the study, acquired and interpreted the data, and drafted the manuscript; H.L. conceived the study and interpreted the data; H.Z. conceived the study, collected data, and revised the manuscript; D.R. critically revised the manuscript and factual content; D.S. conceived and designed the study, acquired data, and critically reviewed the manuscript; Z.Z. performed ultrasound examination, collated related data, and critically reviewed the manuscript; and H.Q. conceived and designed the study, acquired data, and critically reviewed the manuscript. All authors have read and approved the final version of the manuscript.
